# The Neural Circuit Architecture of Social Hierarchy in Rodents and Primates

**DOI:** 10.3389/fncel.2022.874310

**Published:** 2022-05-12

**Authors:** Emanuel Ferreira-Fernandes, João Peça

**Affiliations:** ^1^CNC—Center for Neuroscience and Cell Biology, University of Coimbra, Coimbra, Portugal; ^2^Institute of Interdisciplinary Research (IIIUC), University of Coimbra, Coimbra, Portugal; ^3^Department of Life Sciences, University of Coimbra, Coimbra, Portugal

**Keywords:** social hierarchies, neuronal circuits, dominance, status syndrome, microcircuitry, social status

## Abstract

Social status is recognized as a major determinant of social behavior and health among animals; however, the neural circuits supporting the formation and navigation of social hierarchies remain under extensive research. Available evidence suggests the prefrontal cortex is a keystone in this circuit, but upstream and downstream candidates are progressively emerging. In this review, we compare and integrate findings from rodent and primate studies to create a model of the neural and cellular networks supporting social hierarchies, both from a macro (i.e., circuits) to a micro-scale perspective (microcircuits and synapses). We start by summarizing the literature on the prefrontal cortex and other relevant brain regions to expand the current “prefrontal-centric” view of social hierarchy behaviors. Based on connectivity data we also discuss candidate regions that might inspire further investigation, as well as the caveats and strategies that have been used to further our understanding of the biological substrates underpinning social hierarchy and dominance.

## Introduction

Social status is recognized as a major social determinant of health (Wilkinson and Marmot, 1998). Seminal studies by Marmot and Sapolsky provided evidence that social hierarchies in primates, both human and non-human, lead to social gradients in health, whereby lower rank individuals exhibit progressively higher morbidity and mortality rates (Marmot, 2004; Marmot and Sapolsky, 2014). Examples of this can be found broadly in human societies, for example, in the Scottish city of Glasgow, there is a 28-year difference in life expectancy between the wealthiest and poorest suburbs (Hanlon et al., 2006), and in the poorer neighborhoods of Washington DC, life expectancy decreases by 16 years compared to wealthier areas (Murray et al., 2006). Similar correlations are reported in all rich, poor, and intermediate countries surveyed (Victora et al., 2003; Hurt et al., 2004; Marmot, 2004). Socioeconomic status is, therefore, the single strongest predictor of life expectancy and disease risk in humans (Sapolsky, 2004, 2005). It seems obvious that low-status individuals have limited access to essential resources, partially explaining their health conditions, however, the social gradient in health persists in human populations free from extreme poverty, suggesting that other factors might underlie this phenomenon. In this regard, Marmot and Sapolsky proposed a novel perspective, whereby gradients in psychosocial stressors would contribute to the observed gradient in health, even in the absence of substantial deprivation (Marmot and Sapolsky, 2014). Since their studies focused on humans or troops of baboons, the gradient of stress, due to the respective organization of these societies, tends to increase in subordinates.

Subordinate baboons displayed basal hypercortisolism in the range known to adversely impact blood pressure, insulin sensitivity, and immunity (Sapolsky et al., 2000). This gradient of stress is, however, not universal, and depends on the stability of the hierarchy and the mechanisms used for rank maintenance (Sapolsky, 2005). For example, in highly stable, despotic societies where rank is maintained through intimidation, which is prominent among humans and baboons, subordinate individuals are under pressure. However, in unstable, despotic societies that require frequent physical reassertion of dominance, dominant individuals experience the highest levels of stress. Even though Marmot’s and Sapolsky’s hypothesis is under debate, it calls attention to the relevance of social hierarchies for stress gradients and health in animal societies (Marmot and Sapolsky, 2014).

Since hierarchies inevitably produce inequalities, it is legitimate to ask why natural selection has favored them. Under naturalistic settings, animals have to compete for limited resources, including food, mating opportunities, and nesting sites. To mitigate the risk of constant fighting for limited resources, animals adopted basic, albeit not mutually exclusive strategies, such as territoriality and the formation of social hierarchies (Chase, 1974; Wilson, 1975). When groups of conspecifics are confined to the same territory, hierarchies dictate an individual’s priority in accessing the resources (Wilson, 1975). Dominant individuals have higher reproductive success (Samuels et al., 1984; Ostner et al., 2008; Rodriguez-Llanes et al., 2009) and increased access to food (Whitten, 1983; Isbell et al., 1999) at the expense of sustaining a higher metabolic rate (Røskaft et al., 1986; Martin and Salvador, 1993) and higher propensity for injuries (Røskaft et al., 1986), whereas subordinate individuals save energy and avoid injuries at the expense of lower reproductive success (Martin and Salvador, 1993; Ellis, 1995) and lower access to food resources (Dittus, 1977; Owens and Owens, 1996). Still, subordinate individuals might have ecological value presumably because dominant animals eventually need replacement (Darling, 2008) and because they might abandon their current group to form new groups (Christian, 1970; Esser, 1971). Therefore, territoriality and social hierarchies ensure efficient sharing of resources and regulate aggression. However, there is considerable plasticity in these social strategies, as illustrated by the adaptation of rodents to their population density (Singleton and Krebs, 2007). Under low population densities, as seen in the remaining groups of wild rodents, their territoriality is reinforced, whereas under high population densities, as seen in rodents living alongside human settlements and in lab cages, social hierarchies are reinforced (Singleton and Krebs, 2007).

Natural selection favored a strategy that ensures the fitness of the population at the expense of the individual well-being, so it might be tempting to dismiss social hierarchies in favor of fully egalitarian systems. However, disturbing hierarchies may have a devastating effect on the population, as illustrated by the famous experiments carried out by John B. Calhoun (for a complete historical review, see Ramsden and Adams, 2009). While addressing Malthusian concerns of overpopulation, Calhoun conducted a series of behavioral studies where rats (Calhoun, 1970) and mice (Calhoun, 1973) were bred in a closed environment free from predators and deprivation. Notably, these “Rodent Utopias” resulted in population collapse catalyzed by unconstrained growth, which drastically changed the rodents’ natural behaviors, rendering these societies and the remaining individuals unviable. This catastrophic change in behavior was named “behavioral sink” (Bliss, 1962; Calhoun, 1970). Focusing on Universe 25 (Calhoun, 1973)—Calhoun’s most famous experiment—social collapse was preceded by a profound perturbation of the social hierarchy, noticeable on the 315th experimental day. Omega males, spurned by females and devoid of social duties, wandered apart from the larger groups in a solitary existence, losing social skills (Calhoun, 1973). Alpha males, on the other hand, became highly aggressive, spontaneously attacking other individuals regardless of their gender (Calhoun, 1973). Notably, as dominant males abandoned their traditional roles, generalized aggressiveness was reported to escalate among females in the attempt to defend their nests, resulting also in violence towards their own pups (Calhoun, 1973). These findings were essentially recapitulated in all Universe experiments (Calhoun, 1966, 1970). When raised in such conditions of severe early-life stress, upon reaching maturity, several animals would disregard most social activities and focus only on feeding and grooming (Calhoun, 1977). These so-called “Beautiful ones” could no longer form new societies when placed in a novel enclosure, eventually leading to population extinction in the Universe experiments, well before the maximal putative limit in terms of space, food, and water availability (Calhoun, 1977). This overwhelming experiment was used to illustrate the putative consequences of unconstrained population growth, however, it also showed that the breakdown of social behaviors and hierarchies was the precipitating step in the downward spiral within the enclosed environments. The relevance of social hierarchies becomes even more expressive if one realizes that maladaptive behavior and population dysfunction started significantly before the theoretical maximal population level.

In light of the critical impact of social hierarchies on individual health and ecological balance, efforts have been made to uncover their supporting mechanisms. This question was tackled using neuroendocrine studies, but a fundamental goal in the field has been the delineation of the neural network subserving the encoding, inference, and use of the social rank to inform actions. In this review, we compare and integrate findings from primate (human and non-human), fish, and rodent studies to create a model of the neural network supporting hierarchical behavior. We start with a macroscale (i.e., brain regions) description of that network and further highlight mesoscale (i.e., circuits) and microscale mechanisms (micro-circuits and synapses) that serve the organism in its navigation of social hierarchical behavior. We also discuss the major emerging candidates in these social brain networks.

## The Determinants of Social Hierarchies

**Figure 1:**
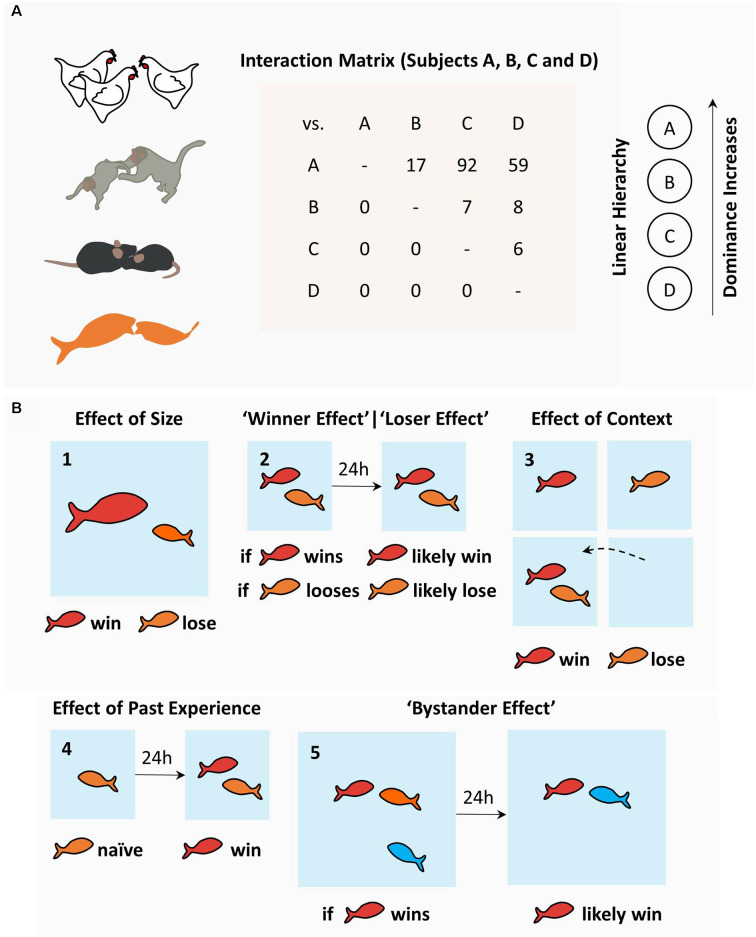
The sociobiology of hierarchies: determinants and consequences. **(A)** Social hierarchies, or pecking orders, emerge in diverse animal species and can be characterized using interaction matrices as used by Schjelderup-Ebbe, depicting the number of attacks (or victories) of one individual over others, which may lead to social hierarchies in a variety of topographies (linear hierarchy displayed from the matrix data). **(B)** Determinants of social hierarchies in fish models include: **(1)** effect of size; **(2)** effect of past winning (“winner effect”) and past losing (“loser effect”); **(3)** the effect of familiar vs. novel context (“homefield”); **(4)** effect of past experience; and **(5)** the bystander effect. Among fish, dominance is attained by bigger fish and winning experience when fighting familiar opponents in a familiar context. Rodents share most of these rules. More complex species, such as primates, have additional, more complex determinants (see text).

Since the description of *pecking orders* in domestic fowls by Schjelderup-Ebbe (Schjelderup-Ebbe, 1922), social hierarchies have been recognized as a near-universal phenomenon among social animals, from insects and fishes to rodents and primates (Figure 1A; Wilson, 1975). Regardless of the species, social hierarchies require contests between conspecifics where several factors, both intrinsic and extrinsic, play a combined and non-linear role in influencing the outcome (Wilson, 1975; Chase et al., 2002). Intrinsic traits include size, age, kinship, gender, and personality traits (Wilson, 1975). Extrinsic factors, on the other hand, are more difficult to isolate and include fatigue, fighting skills, environmental aspects, prior experience, and stochastic events (Wilson, 1975). In rodents, like in many other simple species relying on physical contests, higher ranks are reserved for bigger and less timid males. Size is, therefore, one of the strongest single predictors of outcome in confrontations, where dominant rodents seem to control the space shared by the group (Wilson, 1975). More complex animals, such as primates, have more complex rules. In fact, high-rank primates tend to be descendants of high-rank mothers, bigger, older, and display extroverted behavior (Wilson, 1975). Other critical aspects of understanding of the behavioral and physiological determinants of social hierarchies have also emerged from fish studies, where this field of research is considerably developed (Oliveira, 2005; Hsu et al., 2006; Gonçalves et al., 2017).

**Figure 2:**
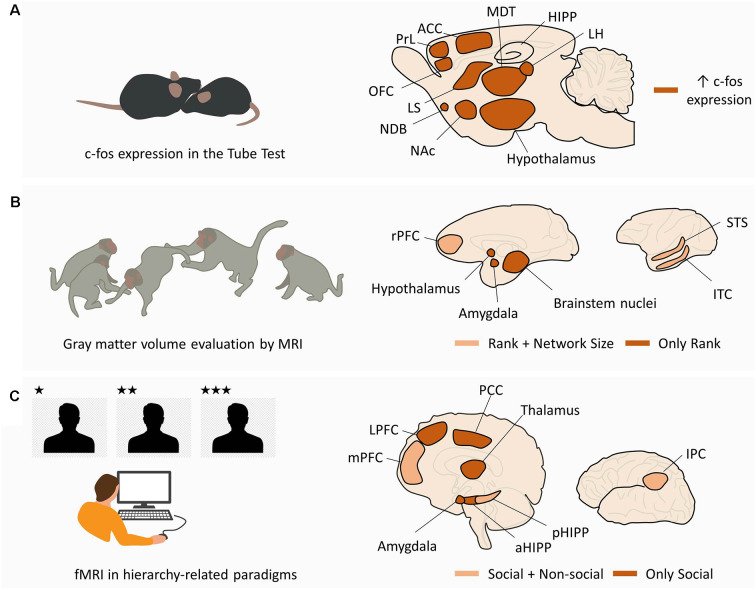
Macroscale networks supporting social hierarchies. **(A)** Macroscale network in rodents. In mice performing tube tests for social hierarchy, c-fos expression in dominant animals is increased in the ventromedial hypothalamus, LH, ACC, medial preoptic area (hypothalamus), and MDT. In addition, pairwise correlations were found between MDT-PrL, MDT-OFC, MDT-ACC, MDT-CA2, ACC-MDT, ACC-LH, ACC-NDB, ACC-LS, and ACC-NAc. **(B)** Macroscale network in non-human primates. MRI studies in non-human primates from different social statuses and living in groups of different sizes have shown a correlation between social rank and gray matter volume in the hypothalamus, amygdale, and brainstem nuclei (“Only Rank”). Gray matter volume in rPFC, STS, and ITC was correlated with both social rank and group size (“Rank + Network Size”). **(C)** Macroscale network in humans. fMRI studies in human subjects performing hierarchy-related tasks have shown selective activation of LPFC, amygdala, aHIPP, thalamus, and PCC by the social component of the task (“Only Social”). The mPFC, pHIPP, and IPC were activated in the social and non-social conditions, suggesting a domain-general function (“Social + Non-social”). See text for other domain-general regions in human literature not depicted in the figure. Abbreviations: LH, lateral habenula; ACC, anterior cingulate cortex; MDT, medial preoptic area (hypothalamus), and mediodorsal thalamus; PrL, prelimbic cortex; OFC, orbitofrontal cortex; NDB, nucleus of the diagonal band; LS, lateral septum; NAc, nucleus accumbens; rPFC, rostral prefrontal cortex; STS, superior temporal sulcus; ITC, inferior temporal cortex; LPFC, lateral prefrontal cortex; aHIPP, anterior hippocampus; pHIPP, posterior hippocampus; PCC, posterior cingulate cortex; mPFC, medial prefrontal cortex; IPC, inferior parietal cortex.

**Figure 3:**
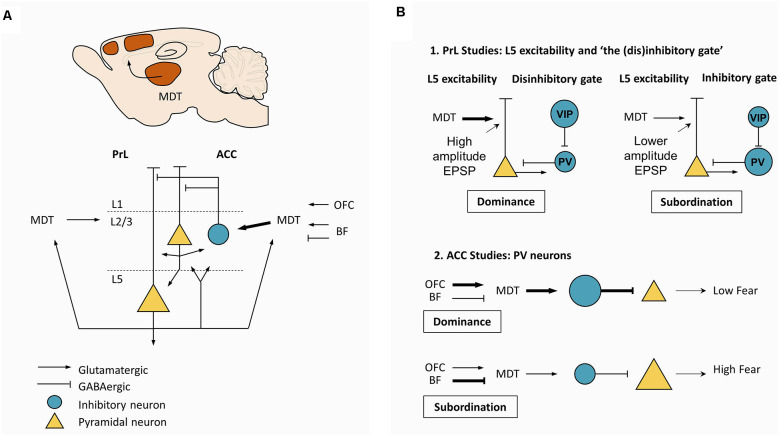
Mesoscale mechanisms supporting dominance. **(A)** Canonical circuit in PrL and ACC. Glutamatergic neurons from MDT project strongly to L2/3 PV neurons in ACC, with a less dense projection to L2/3 in PrL. Depolarization of MDT neurons is determined by excitatory projections from OFC, L5 pyramidal neurons from PrL and ACC, and from excitatory and inhibitory projections from the BF. **(B)** Two major types of studies to explain dominance. **(B-1)** Studies in the PrL support the central role of MDT. Strong excitatory drive coming from MDT is thought to favor effortful, persistent behavior by increasing the excitability in L5 pyramidal neurons. Potentiation of MDT-PrL synapses by repeated winning and activation is thought to increase the probability of winning, triggering a “winner effect”. In addition, a disinhibitory gate may also contribute to regulating the excitability of L5 pyramidal neurons. Specifically, increased activity in the VIP interneurons would open the gate by inhibiting PV interneurons, leading to increased excitability in L5 pyramidal neurons, favoring dominance. On the other hand, increased activity in PV interneurons would close the gate, leading to decreased excitability in L5 pyramidal neurons, favoring subordination. **(B-2)** Studies in the ACC suggest that high MDT excitability and firing due to a strong excitatory drive coming from the OFC would lead to increased depolarization of PV neurons, decreasing fear expression. These hypotheses suggest that differences in synaptic plasticity and neuronal excitability *per se* could explain dominance among rodents. Plasticity and excitability are prone to genetic influences and environmental effects, including hormones and degree of behavioral training. Abbreviations: PrL, prelimbic cortex; ACC, anterior cingulate cortex; MDT, mediodorsal thalamus; PV, parvalbumin; OFC, orbitofrontal cortex; BF, basal forebrain; VIP, vasoactive intestinal peptide.

**Figure 4:**
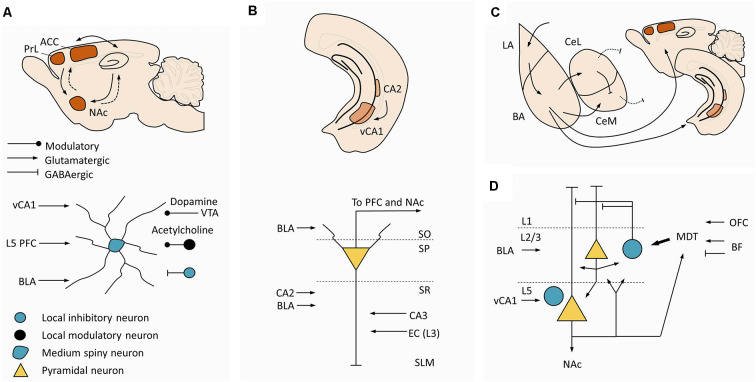
Additional circuits to interrogate in future dominance studies. **(A)** Circuit of the NAc. *Top.* Macroscale circuit. The NAc receives monosynaptic projections (arrows) from PrL, ACC, and ventral hippocampus, projecting back to these regions through indirect connections (dashed arrows). *Bottom.* Canonical microcircuit in the NAc. Medium spiny neurons in NAc are the major targets of L5 pyramidal neurons from PrL and ACC, pointing to striatal circuits as a logical downstream target to expand current ACC and PrL hypotheses for social dominance. **(B)** Circuit in vCA1. *Top.* Macroscale circuit. Hippocampus anatomy highlighting the location of CA2 and vCA1. *Bottom*. Canonical microcircuit in ventral CA1, pyramidal neurons in vCA1 receive inputs from CA2, CA3 and EC, and project to the PFC and NAc. This microcircuit suggests an interplay between dominance mechanisms and the circuits regulating social memory. **(C)** Macroscale circuit in the amygdala. Amygdala anatomy highlighting the connectivity between LA, BA, CeL, and CeM, and the PFC and ventral hippocampus. Arrows represent excitatory projections, blunt lines represent inhibitory projections, and dashed lines represent inhibitory projections to brainstem regions responsible for aggression. **(D)** Microcircuit in the PFC. Panel **(D)** is similar to Figure 3A but here are included afferent projections from vCA1, which target mostly L5 prelimbic neurons (including L5 parvalbumin interneurons), afferent projections from the BLA, which target mostly L2 prelimbic neurons, and efferent projections to the NAc. Other prefrontal subareas receive vCA1 and BLA projections with different laminar distributions compared with the prelimbic region (not illustrated here). This panel summarizes candidate regions upstream and downstream to the prefrontal cortex that may be considered in future studies aiming at unraveling the circuitry supporting hierarchical behavior. Abbreviation: NAc, nucleus accumbens; PrL, prelimbic cortex; ACC, anterior cingulate cortex; PFC, prefrontal cortex; VTA, ventral tegmental area; vCA1, ventral CA1; EC, entorhinal cortex; SO, stratum oriens; SP, stratum pyramidale; SR, stratum radiatum; SLM, stratum lacunosum-moleculare; LA, lateral amygdala; BA, basal amygdala; BLA, basolateral amygdala; CeL, centrolateral amygdala; CeM, centromedial amygdala.

Cichlids and other fish species used in social behavior research readily engage in dyadic contests and form social hierarchies. Behavioral studies have focused mostly on fish size and prior experience to explain social rank (Beaugrand et al., 1991). Not surprisingly, bigger fish tend to conquer higher ranks (Figure 1B-1; but see Beaugrand et al., 1991). Prior experience, on the other hand, has proven to exert a more complex effect on future dyadic contests. In general terms, past victories increase the probability of winning (“winner effect”; Hsu and Wolf, 1999), whereas past losses increase the probability of losing future contests (“loser effect”; Figure 1B-2; Hsu and Wolf, 1999). The detailed analysis of past dyadic encounters and their effect in future contests further isolated: spatial context (Figure 1B-3; Zayan, 1975a; Beaugrand and Zayan, 1985); the opponent’s identity (Zayan, 1974, 1975b; Beaugrand and Zayan, 1985; Madeira and Oliveira, 2017); and past experience (Figure 1B-4; Beaugrand and Zayan, 1985; Beaugrand et al., 1991; Hsu and Wolf, 1999; wining, losing or the absence of fighting experience), as variables with significant meaning. Even though these data were obtained exclusively through dyadic confrontations, winner and loser effects impact the formation of social hierarchies in fish, since randomly chosen winners (and losers) in pairwise contests were more likely to emerge as high ranked (and low ranked) individuals when grouped with conspecifics (Dugatkin and Druen, 2004). Curiously, the effect of prior experience does not necessarily imply overt confrontation. Interestingly, bystanders, who do not engage in overt confrontation but simply observe a contest, also change their winning and losing probabilities in future contests (Figure 1B-5; Johnsson and Åkerman, 1998; Silk, 1999; Oliveira et al., 2001; for review on the “bystander effect” and bystander-related phenomena, see Oliveira, 2005). Similar effects have been described in rodents (Van de Poll et al., 1982; Fuxjager and Marler, 2010), humans (Page and Coates, 2017), and invertebrates (Van Wilgenburg et al., 2010; Stevenson and Schildberger, 2013; Benelli et al., 2015). Efforts have been made to uncover the mechanisms supporting these behavioral observations. A major hypothesis in the field suggests that hierarchical behavior is regulated by the interaction between neuroendocrine factors and neural circuits. While neuroendocrine mechanisms have been thoroughly explored in many animal models (for review, see Oliveira, 2005), the neural circuits supporting social hierarchies are less understood and have been the focus of recent research efforts.

## The Macroscale Networks Supporting Social Hierarchies

Social animals do not just navigate the physical world to survive; they also navigate complex social worlds, where they have to respect hierarchies to maximize benefits, avoid injury and exclusion (Schafer and Schiller, 2018). While neuroendocrine mechanisms certainly shape these behaviors, there is growing interest in uncovering the neural circuits subserving social navigation within hierarchies. In this sense, rodents and primate studies (human and non-human) during hierarchy-related behaviors have been providing a growing list of candidate regions that are modulated and/or necessary for the encoding, inference, and expression of social rank. Although rodent and primate findings do not perfectly overlap, there is considerable evidence toward the existence of a critical network of neural substrates supporting hierarchical behavior (Figure 2).

Large screenings in rodents were performed mostly through the evaluation of a c-fos expression in dominant and submissive mice after performing the tube test (Fan et al., 2019). Compared to submissive mice, dominant animals display significant activity differences in ventromedial hypothalamus, lateral habenula (LHb), anterior cingulate cortex (ACC), medial preoptic area, and mediodorsal thalamus (MDT; Nelson et al., 2019). In addition, nine pairwise correlations significantly differed between dominant and submissive mice, specifically MDT-Prelimbic cortex (PrL), MDT-Orbitofrontal cortex (OFC), MDT-ACC, MDT-CA2, ACC-MDT, ACC-LHb, ACC-Nucleus of the Diagonal Band, ACC-Lateral Septum (LS), and ACC-Nucleus Accumbens (NAc; Nelson et al., 2019; Figure 2A).

There are, however, considerable differences in social behavior between rodents and primates, which raises the question of whether neural substrates differ between species. To tackle this, several structural and functional studies have also been performed in human and non-human primates during hierarchy-related behaviors. Monkeys from different ranks, living in groups of different sizes, exhibit brain structural adaptations observable using structural Magnetic Resonance Imaging (MRI). Specifically, gray matter volume in the amygdala (AMY), brainstem [from the medulla to the midbrain, including parts of the raphe nucleus (RN)], hypothalamus, and basal ganglia (posterior putamen and caudate) were found to be correlated with social rank (Noonan et al., 2014). On the other hand, gray matter volume in the superior temporal sulcus and rostral prefrontal cortex (PFC) was found to be correlated with both social rank and social network size (Sallet et al., 2011; Noonan et al., 2014). Similar structural correlations between gray matter and social network size were also observed in humans, particularly for the AMY (Bickart et al., 2011), OFC (Powell et al., 2012), ventromedial prefrontal cortex (vmPFC; Lewis et al., 2011), superior temporal sulcus (Kanai et al., 2012), and temporal cortex (Kanai et al., 2012; Figure 2B). On a functional domain, human research using hierarchy-related paradigms and functional Magnetic Resonance Imaging (fMRI) has provided compelling evidence of the neural dynamics subserving the encoding, inference, and expression of social rank (Figure 2C). Regarding rank coding, it was found that human subjects generate dissociable neural responses when playing a computer game against (simulated) higher-ranked and lower-ranked players. Individuals playing against a higher rank player displayed increased activity in bilateral occipital/parietal cortex, ventral striatum (vSTR), parahippocampal cortex, and dorsolateral prefrontal cortex (dlPFC), compared to when they faced a lower rank player (Zink et al., 2008). These results were observed in stable hierarchies, where participants were unable to change their rank irrespective of the outcome of the game, and in unstable hierarchies, where the outcome of the game allowed moving up or down in the hierarchy. In the unstable condition, additional brain regions displayed increased activity when participants faced higher rank opponents, including the AMY, medial prefrontal cortex (mPFC), posterior cingulate, bilateral thalamus, primary motor cortex, somatosensory cortex, and supplementary motor area (Zink et al., 2008). However, when individuals repeated the task but were informed that they were playing against computers, dlPFC, AMY, thalamus, posterior cingulate, and mPFC were not significantly activated, suggesting that these brain regions were sensitive to the social component of the task (Zink et al., 2008). In a related work involving the observation of higher and lower ranked individuals, test subjects perceived and gauged the social rank of others. This task engaged the inferior parietal cortex (Chiao et al., 2009).

However, contrary to the paradigms in which ranks are explicitly known *a priori*, in natural conditions, social rank is often inferred from another’s behavior, and demeanor and social hierarchies are progressively assimilated through experience. Functional studies have shed light on the neural substrates supporting rank inference by showing the recruitment of occipitotemporal regions, including the superior temporal, fusiform, and lingual gyri as subjects classified individuals as dominant or submissive based on their facial expressions (Chiao et al., 2008). Moreover, dominance-related signals (brow position, posture, gaze, and gesture) increase the activity in dlPFC and ventrolateral PFC (vlPFC; Marsh et al., 2009). Rank coding and inference presumably allow individuals to acquire knowledge about social hierarchies in order to guide behavior and navigate their social world. The acquisition and use of hierarchy-related information seems to depend upon the AMY, hippocampus (HIPP), and vmPFC (Kumaran et al., 2012). Notably, the AMY and anterior HIPP are specifically engaged when subjects learn and apply knowledge about social hierarchies, compatible with social specificity, whereas the posterior HIPP and vmPFC are engaged in tasks involving the acquisition and use of knowledge about social and non-social hierarchies, compatible with a domain-general role (Kumaran et al., 2012). A parsimonious hypothesis is that social-specific areas (AMY and anterior HIPP) are modulated by rank-related information, further introducing this information in pre-existing domain-general mechanisms subserving domain-general learning, cognitive control, and goal-directed behavior. Spatial navigation can be, however, either egocentric (self-referenced) or allocentric (using distal cues). Interestingly, the acquisition and use of hierarchy-related information seem to show some differences depending on whether one is part of the hierarchy (Self hierarchy) or not (Other hierarchy). Human studies showed that learning Self hierarchies was correlated with the activity in the mPFC, whereas learning both hierarchies (Self and Other) was correlated with the activity in the AMY and HIPP (Kumaran et al., 2016). Curiously, during these studies using Self hierarchies, subjects performed a categorization task where they had to assign pictures of individuals to a pre-learned Self hierarchy and Other hierarchy (Kumaran et al., 2016). Even though this task did not require the explicit retrieval of rank-related information, rank-related activity was detected in the AMY and anterior HIPP when categorizing the pictures and lower-ranked stimuli elicited higher activity (Kumaran et al., 2016). This suggests that social rank becomes an integral trait of each individual in the social network and is spontaneously and implicitly retrieved during social interactions. Rank-related activity was also detected in the mPFC but only when categorizing stimuli from the Self hierarchy (Kumaran et al., 2016).

Despite the value of these findings, the level of variability in the anatomical definition of each region, methodologies, behavioral tasks and results, hinder our ability to fully conciliate all the observations. Still, they provided a preliminary mapping that can now be used to direct more detailed inquiry at the level of circuits, microcircuits, and synapses.

## Mesoscale and Microscale Mechanisms Supporting Social Hierarchies

### Prefrontal Cortex: A Major Hub in Social Hierarchies

The PFC has been consistently suggested as a neural substrate supporting social and hierarchy-related behaviors (Figure 3A), but the mechanisms underlying its role remained unknown until recently. A pioneering study by Hailan Hu and colleagues, found that layer 5 pyramidal neurons in the mPFC play a pivotal role in dominance and submissive behaviors in mice (Wang et al., 2011; Figure 3B-1). Miniature excitatory postsynaptic currents mediated by AMPA receptors in these neurons display increased amplitude, but not frequency, in dominant mice (Figure 3B-1). Moreover, increasing the strengths of these synapses through viral overexpression of AMPA-receptor subunit GluA4 led to dominance behavior, whereas decreasing the strengths of synapses through a viral expression of GluA4 C-tail led to subordinate behavior (Wang et al., 2011). This work stimulated the search for neural pathways capable of acting as molecular switches between dominance and subordinate behaviors. Extending these findings, neuronal populations in mouse mPFC were found to display increased firing rate during effortful behaviors, such as pushing and resistance in the Tube test, but not during retreat, suggesting that mPFC might mediate the behavioral persistence to win the social contest (Zhou et al., 2017). In fact, optogenetic activation (mediated by channelrhodopsin-2, ChR2) and chemogenetic inhibition (mediated by hM4D muscarinic receptors) of mPFC, induced instantaneous winning and losing, respectively, but only when the manipulations were performed in the PL and in rostral ACC (Zhou et al., 2017). In addition, optogenetic stimulation delivered to the mPFC caused the maintenance of dominance behavior and repeated winning in animals experiencing more than six photoinduced wins (Zhou et al., 2017). This effect was inhibited upon treatment with MK801, an antagonist of NMDA receptors, suggesting this effect was due to NMDA-dependent plasticity mechanisms (Zhou et al., 2017). Notably, cingulate parvalbumin-positive interneurons seem to be required for hierarchical behavior as well, since the chemogenetic inhibition of this inhibitory population leads to a pronounced decline in competitive performance among dominant mice, whereas chemogenetic excitation causes a striking increase in competitive performance among subordinate mice (Nelson et al., 2019). More recently, an interneuron-based gating mechanism was described in PFC capable of acting as a switch between dominance and subordination (Zhang et al., 2022; Figure 3B-1). Increased activity of local vasoactive intestinal polypeptide (VIP) interneurons in PFC leads to direct inhibition of local parvalbumin (PV) interneurons, generating a window of opportunity for increased excitability in L5 pyramidal neurons, resulting in dominance. Contrarily, increased activity of local PV interneurons, preceding the activity of VIP interneurons, decreases the excitability of L5 pyramidal neurons, working as a gate and resulting in subordinate behavior, expressed by losing in social confrontations. This work supports previous observations that mice subjected to early life stress develop strong subordinate behavior, which was correlated with morphological alterations in pyramidal neurons in PFC and increased inhibitory postsynaptic current amplitude (Franco et al., 2020). An elegant hypothesis to explain this observation is that early life stress alters the local wiring of PFC, creating conditions to exacerbate the gating mechanism described above (Franco et al., 2020).

### Thalamus: A Driver of the Prefrontal Cortex

Even though there is considerable evidence suggesting the PFC is a central hub in social and hierarchy-related behaviors, the PFC function depends upon the activity of its upstream and downstream regions. This raises the question of which partners interact with the PFC during hierarchical behavior. Since the mPFC receives dense projections from MDT (Hoover and Vertes, 2007; Figure 3A) and since repeated defeat-induced social avoidance leads to depression of MDT-mPFC synapses (Franklin et al., 2017), strengthening of MDT-mPFC synapses could contribute to attaining a high social rank. Indeed, repeated winning in a tube test led to a sustained increase in the field excitatory postsynaptic potentials in MDT-mPFC synapses *in vivo* (Figure 3B-1), and long-term potentiation of MDT-mPFC synapses using ChR2 *in vivo* elicited dominance (Zhou et al., 2017).

Expanding these findings regarding the MDT-mPFC projection, which focused mostly on PrL-targeting axons, the MDT-ACC projection was also found to trigger dominant and subordinate behavior (Figure 3B-2). Chemogenetic excitation of glutamatergic MDT neurons increased competitive performance in subordinate mice, without affecting dominant animals (Nelson et al., 2019). Notably, this glutamatergic population received monosynaptic input from the deep layers of ACC, PrL, and OFC, while projecting to layer 2/3 of ACC, PrL, and OFC, and to the NAc (Nelson et al., 2019). Their major output area was, however, the ACC, where glutamatergic MDT neurons target parvalbumin-positive neurons (Nelson et al., 2019). Whole-cell recordings in these MDT neurons revealed two functional clusters, one with a low firing rate and the other with a high firing rate (Nelson et al., 2019). Dominant mice had an approximately five-fold higher ratio of high vs. low firing rate neurons, suggesting that MDT neurons might have two excitability states according to the animal’s rank. Focusing on the inputs to MDT neurons, dominant mice had a higher frequency and amplitude of spontaneous excitatory postsynaptic currents, whereas submissive animals showed a higher frequency of spontaneous inhibitory postsynaptic currents (Nelson et al., 2019). Notably, OFC was shown to make excitatory synapses in these MDT neurons, while the basal forebrain (BF) makes excitatory and inhibitory synapses (Nelson et al., 2019). ChR2-based paired-pulse stimulation of OFC-MDT synapses produced facilitation in submissive mice and depression in dominant mice, compatible with increased release probability in the latter (Nelson et al., 2019). When the same strategy was applied to BF-MDT synapses, which include inhibitory and excitatory synapses, submissive animals showed paired-pulse depression in inhibitory synapses, compatible with increased release probability, and no changes were seen in dominant mice (Nelson et al., 2019). Excitatory synapses showed depression in both groups. Together, these data converge to a model (Figure 3B-2) where dominant behavior is linked to strengthened excitatory OFC-MDT synapses and increased excitatory drive onto parvalbumin-positive cingulate neurons. Submissive behavior, on the other hand, is linked to strengthened inhibitory BF-MDT synapses and less excitatory drive onto parvalbumin-positive cingulate neurons.

In summary, circuit-based research in hierarchy-related behavior suggests that the PFC contributes to hierarchical behavior, with emphasis on layer 5 pyramidal neurons, and the MDT^Glut^-PrL, OFC-MDT^Glut^-ACC^PV^, and BF-MDT^Glut^-ACC^PV^ are pathways whose modulation can bidirectionally switch between dominance and subordination. In addition to these long-range modulators, a local interneuron-based gate, possibly targeted by these modulatory long-range projections, would create windows of increased (VIP neurons) and decreased (PV neurons) excitability in L5 pyramidal neurons, increasing the tendency for dominant and subordinate-related actions, respectively. mPFC could regulate dominance due to its effect on behavioral persistence or, alternatively, the excitatory drive to parvalbumin-positive cingulate neurons could decrease fear expression, prompting mice to exhibit dominant behavior. These pathways should play a substantial role in the networks outlined in Figure 2. Future detailed mesoscale and microscale studies similar to these will allow the molecular and circuit understanding of other hubs which have not received as wide attention.

## New Directions: Emerging Candidates in Social Hierarchies

### Ventral Striatum: The Major Downstream Target of the Prefrontal Cortex

The successful manipulation of social hierarchies upon modulation of PrL and ACC might have ultimately implied changes in striatal circuits (Zhou et al., 2017; Nelson et al., 2019). PrL and ACC display monosynaptic projections to the striatum (STR), whereas the STR communicates indirectly with the cortex *via* polysynaptic projections (Ferino et al., 1987; Wilson, 1987; Levesque et al., 1996; Smith et al., 2014; Figure 4A-*top*). The monosynaptic projections follow a structural gradient, whereby infralimbic (IL) and ventral PrL project to ventromedial STR, the NAc shell, and the remaining PrL and ACC project to the NAc core, further extending to the dorsomedial caudate-putamen complex (Groenewegen et al., 1997). Furthermore, the STR is particularly active during socially rewarding events (Bault et al., 2011), social status information triggers ventral striatal responses that are modulated by one’s own subjective social status (Ly et al., 2011), and vSTR shows increased activity when participants face higher rank individuals (Zink et al., 2008). Despite this evidence, mechanistic research in striatal circuits in hierarchical behavior has been scarce and fragmented. Socially-housed dominant cynomolgus monkeys display significantly higher expression of D2/D3 receptors in the STR compared to socially-housed subordinate and single-housed individuals (Morgan et al., 2002). No significant changes are seen in subordinate individuals (Morgan et al., 2002). Complementing these data, rodent studies have shown specific metabolic signatures in the NAc of dominant and subordinate mice, with dominant mice exhibiting higher levels of energy-related metabolites under basal conditions (Larrieu et al., 2017). Accordingly, accumbal administration of mitochondrial complex I and II inhibitors prompted subordination (Hollis et al., 2015). Within the NAc, the population of dopamine D1 receptor-containing neurons emerged as a targetable candidate to modulate hierarchical behavior, as these neurons were activated by social competition in dominant animals (Hollis et al., 2015; van der Kooij et al., 2018), D1 receptors blockade decreased dominance (van der Kooij et al., 2018), and dominance was improved upon downregulation of the expression of glucocorticoid receptors in that neural population (Papilloud et al., 2020). Together, these data suggest that the mechanistic studies in prefrontal and thalamic loops should be extended to include striatal circuits and accumbal medium spiny neurons (MSN), at least the D1 receptor-containing population (Figure 4A-*bottom*). While NAc is a major target from PrL and ACC, prefrontal (and amygdalar) action potentials alone may be sufficient to reliably trigger spiking activity in the accumbal MSN, which may require concurrent hippocampal action potentials exerting a gating effect on MSNs (O’Donnell and Grace, 1995; French and Totterdell, 2002). From this observation, functional studies devoted to clarifying the role of the mPFC-NAc or AMY-NAc projections in hierarchical behavior should also include the HIPP (Figure 4A-*bottom*). As a final note, the available evidence strongly supports the NAc as an emerging hub in hierarchical behavior, but other striatal divisions might be implied as well. In fact, studies in monkeys found a negative correlation between gray matter volume in the posterior putamen and caudate and social rank (Noonan et al., 2014), suggesting a wider approach to striatal circuits in social and hierarchy-related behaviors.

### Hippocampus: The Social Memory Hub

Our current understanding of hippocampal function results from two major lines of research. Human studies found marked anterograde amnesia for episodic events in patients harboring hippocampal lesions, leading to the conclusion that HIPP is necessary for episodic memory (Scoville and Milner, 1957; Penfield and Milner, 1958; Squire, 2009). On the other hand, rodent research described hippocampal neurons whose firing rate is modulated by the animal’s spatial position, the so-called place cells (O’Keefe and Dostrovsky, 1971; O’Keefe, 1976; O’Keefe and Conway, 1978; O’Keefe, 1979). By firing at particular places, place cells presumably contribute to an internal representation of the environment, a cognitive map (Tolman, 1948; O’Keefe and Dostrovsky, 1971; O’Keefe, 1991). Accordingly, hippocampal inactivation impairs allocentric navigation in rodents (Morris et al., 1982) and these findings were also reproduced in humans (Ekstrom et al., 2003; Parslow et al., 2005), implying that HIPP is necessary for spatial navigation and goal-directed behavior. While efforts were made to conciliate these perspectives (see Eichenbaum and Cohen, 2014), many studies described hippocampal modulation by non-spatial variables, including objects (Manns and Eichenbaum, 2009), time (Eichenbaum, 2014), and social variables (Tavares et al., 2015), providing evidence against a purely spatial theory of the HIPP. Since the observation that conspecifics and other social variables modulate hippocampal neurons (Danjo et al., 2018; Omer et al., 2018), the HIPP has been hypothesized as a hub in social behavior, presumably mapping the animal’s social space (Tavares et al., 2015), and studies showed that the HIPP is necessary for social recognition memory (Okuyama et al., 2016).

Anatomical studies described prominent direct and indirect prefrontal-hippocampal loops (Eichenbaum, 2017). Furthermore, stress prior to a social encounter leads the stressed animal to a long-term subordinate behavior, presumably due to amplification of memory for the hierarchy (Cordero and Sandi, 2007). This effect is blocked by protein synthesis inhibitors and is not evoked by stress alone or, crucially, when the stressed animal faces a novel male, suggesting a superimposed role for recognition memory, for which the HIPP is necessary (Cordero and Sandi, 2007). Curiously, social memory depends upon CA2 (Hitti and Siegelbaum, 2014) and ventral CA1 (Okuyama et al., 2016), and there is a correlation between c-fos expression in MDT and CA2 that changes according to the mouse rank in the Tube test (Nelson et al., 2019). Together, these findings suggest a putative role for hippocampal circuits not only in social behavior but specifically in hierarchy-related behavior.

Siegelbaum and colleagues pioneered the mechanistic study of CA2 in social memory. Indeed, CA2 inactivation produces striking deficits in social memory in a transgenic mouse model (Hitti and Siegelbaum, 2014). Since this publication, CA2 was found to contain a high proportion of social-sensitive cells and CA2 inactivation during social tasks inhibited the formation of social memories (Azahara et al., 2020). As CA2 has restricted output regions (Cui et al., 2013), ventral CA1 (vCA1) has been hypothesized as an intermediate region supporting most of the CA2 communication with cortical and subcortical targets (Meira et al., 2018). Accordingly, studies reported social memory deficits upon inactivation of ventral CA1 (Okuyama et al., 2016) and ventral CA1 projections targeting the NAc (Okuyama et al., 2016) and the PFC (Phillips et al., 2019; Sun et al., 2020). In addition, CA2 inactivation decreases aggressive behavior, illustrated by a marked decrease in the tendency of a resident mouse to engage in social contests with never-met intruders, due to CA2 projections to the LS (Leroy et al., 2018). In light of these, CA2-vCA1-PFC and CA2-vCA1-NAc pathways should be investigated in hierarchical behavior (Figure 4B). Interestingly, hippocampal circuits produce sharp wave ripples (SWR) under native conditions (Buzsáki, 2015) and these were shown to naturally induce plasticity changes (Sadowski et al., 2016), participating in memory consolidation (Ego-Stengel and Wilson, 2010). It would be relevant to assess if SWR could evoke plasticity changes in prefrontal layer 5 pyramidal neurons by strengthening the vCA1-PFC synapses and whether this mechanism would impact the social rank and hierarchical behavior. In light of the recent findings of an interneuron-based gating mechanism for dominance and subordination in PFC (Zhang et al., 2022), it would be also relevant to test whether vCA1 can modulate the gate since there is a well-known projection from vCA1 to the PV interneurons in PFC, whose inhibition produces deficits in social memory (Sun et al., 2020). It would be expected that excitation of PV interneurons in PFC by the same pathway would modulate hierarchical behavior, specifically leading to subordination, establishing a definitive link between hierarchical behavior and social memory, thus explaining the former observation that stress prior to a social encounter leads to long-term subordinate behavior, due to amplification of memory, or, more specifically, due to a putative increase in vCA1-triggered excitation of PV interneurons in PFC.

### Amygdala: An Understudied Area With Diverse Roles in Hierarchy-Related Behavior

The AMY is a classical hub that is broadly implicated in social behavior (Adolphs, 2010). Also, the studies reviewed above have provided strong evidence supporting its specific role in hierarchical behavior in human and non-human primates. In brief, correlational studies found AMY differential activation in human participants during hierarchy-related paradigms, particularly when hierarchies had a social component (Zink et al., 2008; Kumaran et al., 2012, 2016), firing rate modulation of AMY ensembles in monkeys according to the social status of the observed conspecifics (Munuera et al., 2018), and a positive correlation between gray matter volume in the AMY and social status in monkeys, independently of social network size (Noonan et al., 2014). These correlative studies were complemented by lesion studies, but the results are hard to conciliate. In fact, previously dominant monkeys were shown to lose social status and acquire subordinate behavior upon lesions in the AMY (Rosvold et al., 1954), and rats harboring AMY damage lose 85% of the encounters when competing for food against controls (Lukaszewska et al., 1984), suggesting that AMY lesions decrease dominance. Contrary to these, selective ablation of the AMY was found to increase confidence in social interactions (Emery et al., 2001) and increase dominant posture in rhesus monkeys (Machado and Bachevalier, 2006). This heterogeneity might result from variability across lesions and from functional differences between individual AMY subnuclei, but, most importantly, highlights our need for refined mechanistic studies. Surprisingly, this gap remains unexplored, contrasting with the overwhelming progress achieved in the last years regarding AMY research, particularly in classical conditioning (Ehrlich et al., 2009; Orsini and Maren, 2012; Duvarci and Pare, 2014; Tovote et al., 2015). Among the few molecular studies in hierarchical behavior, one group found a positive correlation between social rank and the expression of corticotropin releasing factor mRNA in the AMY of mice (So et al., 2015). Notably, no differences were found when the c-fos expression was compared across dominant and submissive mice after performing the tube test, although c-fos expression was increased in both groups compared to controls, which traversed the tube, but that did not perform the tube test nor engaged in social interactions inside the tube (Nelson et al., 2019). Additional mechanistic studies are clearly needed and two candidate pathways would be a rational starting point, namely the basolateral amygdala (BLA)-mPFC and the BLA-vCA1 synapse (Figures 4C,D). In fact, modulation of the BLA-mPFC projection bidirectionally modulates social behavior in a resident-intruder paradigm (Felix-Ortiz et al., 2016). However, this study used juvenile intruders to avoid aggression, hindering our ability to clearly translate their findings to hierarchical behavior. Nonetheless, optogenetic stimulation of BLA-mPFC projections decreased social behavior, including chasing and contact, and had an anxiogenic effect, whereas inhibition produced the opposite results (Felix-Ortiz et al., 2016). Similar results in terms of social and anxiety-related behaviors were obtained when the same experiments were carried out in BLA-vCA1 projections (Felix-Ortiz et al., 2013). The antisocial and anxiogenic effect of BLA stimulation would suggest a role in subordinate behavior. An alternative explanation was put forward by a growing body of literature which has correlated AMY divisions and aggression (reviewed in Haller, 2018). Still, a putative role for the AMY in aggression does not fully explain its role in hierarchical behavior because dominance does not strictly imply aggression (see Wang et al., 2014). This is clearly illustrated in two studies showing that dominant mice in the tube test do not appear to be more aggressive (Benton et al., 1980), and treatment with cannabinoids decreased aggression and increased winning in the tube test (Masur et al., 1971).

### Subcortical Nuclei and Brainstem Nuclei: The Aggression Circuit

Contrasting with the human studies reviewed above, studies in non-human primates (Noonan et al., 2014) and rodents (Nelson et al., 2019) found a strong engagement of additional subcortical and brainstem nuclei during hierarchical behavior, particularly the hypothalamus, periaqueductal gray (PAG), LHb, LS, and RN.

Among these brain areas, the hypothalamus, PAG, LHb, and LS were mostly studied in the context of aggressive behavior. Their modulation in hierarchical behavior thus suggests a putative overlap between the circuits supporting aggressive behavior and those supporting social hierarchies. The neural substrates of aggression have been reviewed elsewhere (Aleyasin et al., 2018; Flanigan and Russo, 2019) and a considerable amount of data highlighted the hypothalamus as a major hub in aggressive behavior (Hashikawa et al., 2017). In fact, stimulation of its mediobasal aspects, including the mediobasal hypothalamus in cats, the attack area in rats, and the ventrolateral region of the ventromedial hypothalamus in mice, triggered intraspecific aggression in different animal models (Kruk, 1991; Siegel et al., 1999; Lin et al., 2011). Despite the central relevance of the hypothalamus, research on aggression moved beyond the hypothalamus to identify its downstream and upstream partners. The PAG represents the most likely relay between the hypothalamus and the spinal cord (Beart et al., 1988; Chung et al., 1990; Canteras et al., 1994), and has been implicated in social and non-social behavior, including in defense-related behavior like immobility, flight, and escape jump (Wang et al., 2015; Motta et al., 2017). Since aggression must be under efficient control, the hypothalamus and PAG are modulated by upstream brain areas. To illustrate this, the electrical stimulation of AMY divisions was shown to modulate the efficacy of stimulations directly delivered to the mediobasal hypothalamus and PAG to trigger defensive rage in cats. More specifically, stimulation of the basal and medial AMY positively modulated defensive rage, whereas stimulation of the central AMY negatively modulated defensive rage (Shaikh and Siegel, 1994; Shaikh et al., 1994; Siegel et al., 1999). While no such studies were carried out in rodents, rivalry-aggression in the resident-intruder test reliably increased c-fos expression in medial AMY, BLA, and cortical AMY, with mild to no effect in central AMY (Halász et al., 2002; Veening et al., 2005; Duncan et al., 2009; Konoshenko et al., 2013). Although the hypothalamus-PAG circuit is a canonical hub in aggressive behavior, efforts have been made to find additional partners and new ones are emerging, specifically LHb, LS, RN, and even the mPFC (see Aleyasin et al., 2018). Aggressive, but not non-aggressive, mice develop conditioned place preference toward a context where they confronted a subordinate intruder (Golden et al., 2016). Optogenetic inhibition of GABAergic BF-LHb projections in aggressive males abolishes conditioned place preference, whereas its stimulation in non-aggressive males induced conditioned place preference (Golden et al., 2016). These manipulations did not affect the initiation of aggressive behavior, suggesting that GABAergic BF-LHb projections bidirectionally control the valence of aggression. On the other hand, optogenetic stimulation of the mPFC and LS decreased inter-male aggression (Takahashi et al., 2014), but stimulation of LS-projecting CA2 neurons increased inter-male aggression in mice (Leroy et al., 2018). The studies reviewed in this section can hardly illustrate all the exciting advances in the field of aggressive behavior but clearly show that hierarchical and aggressive behavior are indissociable, albeit distinct, and it is crucial to address the channels supporting the cross-talk between the two networks.

Focusing on the RN, MRI studies showed a positive correlation between gray matter volume in the RN, the major source of serotonin in the brain, and social dominance in monkeys (Noonan et al., 2014). Strengthening these data, fluoxetine treatment enhances serotonergic signaling in vervet monkeys, leading to decreased aggression, more affiliative behavior, and better social skills, resulting in higher social rank (Raleigh et al., 1991). On the other hand, monkeys with low serotonergic signaling display impulsive aggression and lower social rank (Raleigh et al., 1991). This association between low serotonergic signaling, impulsive behavior, and aggression was recapitulated in other species, including rodents (Caramaschi et al., 2007; Audero et al., 2013) and fish (Winberg and Nilsson, 1993; Cubitt et al., 2008). While these studies suggest a reproducible negative association between serotonin levels and aggression, the relation between serotonergic signaling and dominance is more complex, probably due to the skills needed to attain high social ranks, which vary from species to species depending on their social complexity and the role played by aggression in the hierarchy. In this sense, increasing the serotonin levels in vervet monkeys resulted in higher social ranks probably because it enhanced the social skills necessary to navigate their hierarchies and invest in affiliative interactions. While human studies supporting a role for serotonin in social dominance are still sparse, treatment with antidepressant medications or serotonin precursors, which increase serotonin levels, also increased the frequency of dominant behaviors (Moskowitz et al., 2001). However, for simpler animals whose social ranks strongly depend upon their agonistic behavior, a similar increase in serotonergic signaling and decreased aggression would be a disadvantage. To illustrate this, pharmacological inhibition of serotonergic activity caused a shift from subordination to dominance in rats competing for water, whereas stimulation of the serotonergic system had the opposite effect (Kostowski et al., 1984). In addition, subordinate fish consistently show high serotonergic activity, in association with decreased locomotor activity, decreased aggression, and lower food intake (Winberg and Nilsson, 1993; Cubitt et al., 2008). Curiously, serotonergic signaling in rodents also showed a sex-dependent effect on dominance, since hypothalamic injection of 8-OH-DPAT, a serotonin 1A agonist, decreased aggression in male hamsters, but increased the aggression level in female hamsters (Terranova et al., 2016). Together, these findings suggest that the PFC-RN loop would be an interesting candidate pathway to target in hierarchical behavior. Effortful behavior has been studied in the rat using a forced swim test, where animals display epochs of resilient behavior in attempting to escape the water tank, interchanged with epochs of immobility in which they stop struggling (Warden et al., 2012). Notably, optogenetic stimulation of RN-targeting mPFC axons triggers resilient escaping behavior, whereas direct stimulation of the RN only increases general locomotor activity (Warden et al., 2012). It is tempting to speculate that PFC-RN projections would be implicated in resilience during stress and that serotonergic activity contributes to coping mechanisms within social settings (for a more detailed review on neuromodulation and social hierarchies, see Watanabe and Yamamoto, 2015; Qu et al., 2017).

## Current Caveats and Future Strategies to Investigate Hierarchical Behavior

The combination of different animal models and technologies has provided a considerable understanding of the neural substrates supporting social hierarchies, from the identification of macroscale regions engaged to the mechanistic dissection of some prominent cell-specific hubs. Despite the evidence reviewed here, the core questions in the field remain largely unanswered. Finding neural correlates (structural or functional) during hierarchical behavior is an important exploratory strategy to quickly identify candidate regions, including a dissection of what brain areas are necessary and sufficient for a given behavior. However, this is still distant from a complete dissection of the neural basis and computations underlying the phenomenon. To complement this strategy the following strategies could prove beneficial:

### Investing in *In vivo* Recordings in Freely-Behaving Animals to Understand Intraregional and Interregional Mechanisms

To understand the neural mechanisms supporting social hierarchies it is essential to record neural activity in multiple brain regions, while animals perform hierarchical behaviors. This will provide access to the populational dynamics, allowing the study of the neural ensembles engaged during hierarchical behavior, their relations in upstream and downstream brain areas, and their relation with the brain rhythms that organize populational activity (Buzsáki, 2010; Buzsáki and Watson, 2012). *In vivo* recordings can be complemented by modern genetic models with conditional expression of neuronal markers to identify neural ensembles active during hierarchical behavior (Reijmers et al., 2007; Liu et al., 2012; Kim and Cho, 2017), characterize them in terms of cell types, and tag neurons during *in vivo* recordings (Tanaka et al., 2018). Besides fostering an understanding of neural mechanisms, this will provide an integrative approach whereby neural mechanisms underlying hierarchical behavior might be integrated with domain-general mechanisms common to other social and non-social behaviors (Schafer and Schiller, 2018; Ramsey and Ward, 2020).

### Improve Manipulation Studies

Modern optogenetic (Fenno et al., 2011; Lee et al., 2020) and pharmacogenetic (Roth, 2016) tools revolutionized neurosciences by allowing the reversible manipulation of genetically defined neural populations. However, direct application of these tools to stimulate and inhibit brain regions during behavior can identify regions of potential interest, but fails to provide mechanistic insight and may even lead to epiphenomena that occlude naturalistic behavior. Efforts should be made to combine manipulations with *in vivo* recordings to monitor the changes produced in the populational dynamics. Furthermore, optogenetic- and pharmacogenetic-based stimulation (unlike inhibition) should be interpreted with caution as these methods do not preserve native brain dynamics. This may be mitigated by adopting new tools that increase neuronal excitability without triggering action potentials.

### *In vivo* Recordings and Manipulations in Non-human Primates

When studying social behavior and social hierarchies, there are considerable differences in the intrinsic and extrinsic factors governing social rank in rodents and primates. In addition, major substrates of hierarchical behavior as the PFC, specifically its lateral division, do not have a rodent homolog so they can only be properly studied using non-human primates (Carlén, 2017). In this sense, efforts should be made to translate rodent protocols to non-human primates. Notably, small non-human primates are emerging models in Neurosciences that have the potential to open new avenues in the study of the neural substrates of social behavior and associated disorders (Miller et al., 2016; Feng et al., 2020).

## Conclusion

Social hierarchies have profound implications for species’ survival and individual health, but the neural mechanisms subserving hierarchical behavior remain elusive, hindering our ability to signal dominance- or subordination-prone individuals and to design strategies to mitigate hierarchy-related effects on health. Correlative and manipulation studies in human and non-human primates and rodents led to the identification of distributed brain networks underpinning hierarchical behavior, as well as mechanisms whose modulation bidirectionally switches between dominant and subordinate phenotypes in animal models. In this review, we synthesized the available knowledge and models of networks supporting social hierarchies. However, additional *in vivo* monitoring of neural activity across multiple brain regions is needed to foster the understanding and integration of how information flow across candidate regions acts in the service of social hierarchy behavior.

## Author Contributions

EF-F and JP wrote the manuscript. JP directed the bibliography search and defined the scope of the review. All authors contributed to the article and approved the submitted version.

## Conflict of Interest

The authors declare that the research was conducted in the absence of any commercial or financial relationships that could be construed as a potential conflict of interest.

## Publisher’s Note

All claims expressed in this article are solely those of the authors and do not necessarily represent those of their affiliated organizations, or those of the publisher, the editors and the reviewers. Any product that may be evaluated in this article, or claim that may be made by its manufacturer, is not guaranteed or endorsed by the publisher.

## Abbreviations

ACC: Anterior cingulate cortex
aHIPP: Anterior hippocampus
AMY: Amygdala
BA: Basal amygdala
BF: Basal forebrain
BLA: Basolateral amygdala
CeL: Centrolateral amygdala
CeM: Centromedial amygdala
dlPFC: Dorsolateral prefrontal cortex
EC: Entorhinal cortex
fMRI: Functional magnetic resonance imaging
HIPP: Hippocampus
IL: Infralimbic cortex
IPC: Inferior parietal cortex
ITC: Inferior temporal cortex
LA: Lateral amygdala
LHb: Lateral habenula
LS: Lateral septum
MDT: Mediodorsal thalamus
mPFC: Medial prefrontal cortex
MRI: Magnetic resonance imaging
MSN: Medium spiny neurons
NAc: Nucleus accumbens
NDB: Nucleus of the diagonal band
OFC: Orbitofrontal cortex
PAG: Periaqueductal gray
PCC: Posterior cingulate cortex
PFC: Prefrontal cortex
pHIPP: Posterior hippocampus
PrL: Prelimbic cortex
PV: Parvalbumin
RN: Raphe nucleus
rPFC: Rostral prefrontal cortex
SLM: Stratum lacunosum-moleculare
SO: Stratum oriens
SP: Stratum pyramidale
SR: Stratum radiatum
STR: Striatum
STS: Superior temporal sulcus
SWR: Sharp wave ripples
vCA1: Ventral CA1
VIP: Vasoactive intestinal polypeptide
vlPFC: Ventrolateral prefrontal cortex
vmPFC: Ventromedial prefrontal cortex
vSTR: Ventral striatum
VTA: Ventral tegmental area.
